# Supplementary material for: Effect of Central Obesity and Hyperandrogenism on Selected Inflammatory Markers in Patients with PCOS: A WHtR-Matched Case-Control Study
Source: J Clin Med. 2020 Sep 20;9(9):3024. doi: 10.3390/jcm9093024 (PMC7565377; doi:10.3390/jcm9093024)
Supplement: Supplementary file 1 [file jcm-09-03024-s001.pdf]

Supplement

Table S1. Limits of detection of assessed hormones (electrochemiluminescence (ECLIA), Cobas 6000 equipment, Roche Diagnostics, Switzerland).

Hormone	Limit of detection
AMH (pmol/L)	0.214
DHEAS (μg/dL)	0.100
E2 (pg/mL)	5
FSH (mIU/mL)	0.100
Insulin (μIU/mL)	0.2
LH (mIU/mL)	0.100
SHBG (nmol/L)	0.350
T (nmol/L)	0.087

AMH—Anti-Müllerian hormone; DHEAS—dehydroepiandrosterone sulfate; E2—estradiol; FSH—follicle-stimulating hormone; LH—luteinizing hormone; SHBG—sex hormone binding globulin; T—total testosterone.

Table S2. Baseline anthropometric findings in the PCOS patients and CON groups.

	**PCOS** n = 270	**CON** n = 125	p value
Age	24.83 (6.67)	26.13 (7.25)	0.02
Weight (kg)	65.00 (19.50)	62.00(15.00)	0.003
Height (cm)	167.00 (8.00)	167.00 (7.00)	NS
BMI (kg/m^2)	23.46 (7.70)	22.04 (5.68)	0.001
WC (cm)	77.00 (19.00)	73.50 (14.00)	0.04
HC (cm)	88.00 (17.00)	85.50 (12.50)	NS
WHR (-)	0.88 (0.10)	0.88 (0.10)	NS
WHtR (-)	0.46 (0.12)	0.44 (0.09)	0.04

BMI— body mass index; HC - hip circumference; WC—waist circumference; WHR—waist to hip ratio; WHtR—waist to height ratio.

Data are presented as median ± interquartile range. There was used nonparametric Mann-Whitney U test. $p < 0.05$ was considered to be statistically significant
NS—not statistically significant

Table S3. Biochemical parameters in the PCOS and CON groups.

	**PCOS** n = 270	**CON** n = 125	p value
Glucose 0' (mg/dL)	87.00 (9.00)	88.00 (10.00)	NS
Insulin 0' (mIU/mL)	9.06 (7.00)	8.73 (6.05)	NS
HOMA-IR (-)	2.01 (1.77)	1.85 (1.51)	NS
Glucose 120' (mg/dL)	94.00 (28.00)	95.00 (29.00)	NS
Insulin 120' (μIU/mL)	42.03 (35.80)	39.10 (30.60)	NS
TC-C (mg/dL)	174.50 (34.00)	173.00 (39.00)	NS
LDL-C (mg/dL)	90.60 (35.80)	85.20 (33.50)	NS
TG-C (mg/dL)	73.00 (46.00)	75.00 (40.00)	NS

HDL-C (mg/dL)	64.00 (21.00)	71.00 (23.00)	NS

HDL-C—high density lipoprotein cholesterol; HOMA-IR—homeostasis model assessment for insulin resistance index; LDL-C—low density lipoprotein cholesterol; TC-C—total cholesterol; TG-C—triglycerides.

Data are presented as median± interquartile range. There was used nonparametric Mann-Whitney U test. $p<0.05$ was considered to be statistically significant
NS—not statistically significant

Table S4. Hormonal parameters in the PCOS and CON groups.

	**PCOS** *n* **= 270**	**CON** *n* **= 125**	*p* **Value**
FSH (mIU/mL)	5.80 (2.10)	5.60 (3.40)	NS
LH (mIU/mL)	8.70 (8.40)	6.25 (6.60)	<0.001
DHEAS (μg/dL)	296.50 (154.00)	251.00 (160.00)	0.002
E2 (pg/mL)	42.00 (37.50)	47.00 (58.00)	NS
T (nmol/L)	1.70 (1.00)	1.20 (0.80)	<0.001
SHBG (nmol/L)	57.60 (42.70)	59.65 (65.00)	0.007
FTI (%)	3.15 (2.76)	2.00 (2.40)	<0.001
A (ng/mL)	4.01 (2.27)	2.81 (1.79)	<0.001
AMH (pmol/L)	51.98 (36.79)	26.37 (18.45)	<0.001

A—androstendione; AMH—*Anti-Müllerian hormone*; DHEAS—*dehydroepiandrosterone sulfate*; E2—estradiol; FSH—*follicle-stimulating* hormone; FTI—free testosterone index; LH—*luteinizing hormone*; SHBG—sex hormone *binding* globulin; T—total testosterone.

Data are presented as median± interquartile range. There was used nonparametric Mann-Whitney U test. $p<0.05$ was considered to be statistically significant
NS - not statistically significant

Table S5. Inflammatory parameters for PCOS and CON groups.

	**PCOS** *n* **= 270**	**CON** *n* **= 125**	*p* **value**
hsCRP (mg/L)	0.80 (1.60)	0.70 (2.10)	NS
WBC x10^3/uL	6.11 (2.29)	5.83 (2.10)	NS
MHR (-)	6.95 (3.96)	6.71 (4,27)	NS
LMR (-)	4.30 (1.84)	4.14 (2.08)	NS

hsCRP—high sensitivity C-reactive protein; LMR—lymphocyte to monocyte ratio; MHR—monocyte-to-high-density lipoprotein cholesterol ratio; WBC—white blood cell count.

Data are presented as median± interquartile range. There was used nonparametric Mann-Whitney U test. $p<0.05$ was considered to be statistically significant
NS—not statistically significant
